# Analysis of Bone Density and Bone Morphometry by Periapical Radiographs in Dental Implant Osseointegration Process

**DOI:** 10.1155/2023/4763961

**Published:** 2023-04-03

**Authors:** Ratih Trikusumadewi Lubis, Azhari Azhari, Farina Pramanik

**Affiliations:** ^1^Dentomaxillofacial Radiology Specialist Program, Faculty of Dentistry, Padjadjaran University, Bandung, Indonesia; ^2^Department of Dentomaxillofacial Radiology, Faculty of Dentistry, Padjadjaran University, Bandung, Indonesia

## Abstract

**Objective:**

This research aimed to analyze the differences in bone density and bone morphometry by periapical implant radiography in the dental implant osseointegration stages.

**Methods:**

This experimental research uses 12 periapical radiographs of tibial bones from a New Zealand white rabbit (*Oryctolagus cuniculus*). The analysis was performed on day 3, 14, and 28 of the osseointegration stages with density, trabecular thickness (Tb.Th.), trabecular separation (Tb.Sp.), and trabecular number (Tb.N.) as parameters. The implant used is a titanium alloy and coated by SA (sunblasted with alumina acid) of 4 mm in diameter and 7 mm in length. The radiographic assessment of the osseointegration process is obtained with the region of interest (ROI) segmentation results. Additionally, each ROI was analyzed for bone density and morphometry using the open-source ImageJ software with the BoneJ plugin. The significant difference was evaluated by analysis of variance (*F*-test) with *p* < 0.05 and nonparametric Kruskal–Wallis test with *p* < 0.05.

**Results:**

Analysis of the osseointegration images of dental implants at day 3, 14, and 28 with the periapical X-ray modality shows significant differences (*p* < 0.05) in the parameters measuring density and trabecular thickness (Tb.Th.). In the variables of trabecular separation (Tb.Sp.) and number (Tb.N.) (*p* > 0.05), there is no significant difference.

**Conclusion:**

Based on the results, density and trabecular thickness (Tb.Th.) showed a significant difference between healing times. However, trabecular separation (Tb.Sp.) and trabecular number (Tb.N.) showed no difference in healing time.

## 1. Introduction

A dental implant is a biocompatible material that is implanted in the mandibular or maxillary bone as a substitute for tooth root function and provides additional support for a dental prosthesis [1, 2]. The success of a dental implant depends on its ability to gradually integrate with the surrounding tissues, which is called osseointegration [3, 4]. The basic success criteria of the dental implant are immobility or interrelationship of the various components such as implant material, bone quality of recipient, surgical technique, absence of infection and peri-implant diseases, and adequate width of the attached gingiva [5, 6].

Osseointegration is the direct contact of the bone with the implant surface without the presence of a fibrous tissue layer [7]. The process is largely determined by the early stages of the bone healing process, including the inflammation, proliferation, and remodeling phases, and many factors influence the formation and maintenance of bone at the implant surface [8, 9]. The main goals of the inflammatory phase are to remove dead tissue and prevent colonization and infection by pathogenic microbial agents, and these phases become the most important. Subsequently, this phase begins immediately after the trauma until the 5th posttraumatic day [10] and can even last up to the 2nd week [8]. In addition, the proliferative or repair phase balances the formation and regeneration of scar tissue [11, 12], and it is characterized by forming soft and hard callus and osteoid [13] secretion, which lasts from day 3 to 14 [12]. The final phase is remodeling from day 21 to about 1-year postinsertion [12]. The remodeling is referred to as secondary bone formation, which is the last phase to improve the shape, structure, and mechanical properties of bones and improve the stability of the implant placement area [12, 13].

Bone density is one assessment of its quality that affects the success rate of the osseointegration process [14]. Bone density describes the relative size of marrow space in a unit of bone and it is directly proportional to the primary stability of a dental implant [15]. Bone morphometric parameters are also used to observe changes in the structure in more detail to describe the changes in the osseointegration process. Based on the American Society for Bone and Mineral Research (ASBMR), several structural parameters are used to represent the architecture of trabecular bone, namely, trabecular number (Tb.N.), trabecular thickness (Tb.Th.), and trabecular separation (Tb.Sp.) [16–18]. Various modalities can be used in the assessment of osseointegration, with the reference in the assessment of the accuracy and density being the modality of computed tomography (CT) [19]. Cone-beam computed tomography (CBCT) enables the visualization of high-contrast structures of the oral region (bone, teeth, air cavities) at a high resolution [20]. However, several limitations of CBCT such as high radiation exposure, high cost, limited accessibility, and can display of some noise, scatter, or cupping artifacts must be considered by the dentist [21].

Periapical radiographs are one of the most widely used modalities for planning, preoperative evaluation, and minor oral surgical procedure [22]. These radiographs are also often used to examine or analyze a single dental implant in the edentulous jaw area [23]. In addition, periapical radiographs have fairly good resolution and detail, at least twice as much as extraoral radiographs, have low radiation exposure, are inexpensive, and are easily set up and used [24]. These radiographs can determine the approximate height of the alveolar bone, the distance between the implant site and the anatomical structure, and the quality of the alveolar bone by looking at the trabecular pattern around the implant [25].

A quantitative method for analyzing periapical radiographs is the measurement of grayscale variation [26]. The mean grayscale value is directly proportional to the bone density value [27]. In the inflammatory stage, the radiographic density is not visible because the implant interface zone is occupied by a transient matrix rich in collagen fibrils and blood vessels [28]. This results in low bone density early in implant placement, evidenced by a radiolucent appearance on radiographs [29]. The radiograph at the proliferation stage showed a slightly increasing in density and the radiograph appearance will be an intermediate radio into radiopaque along with the healing process [30]. However, the stages of radiography are not understood due to limitations such as those provided only on the two-dimensional image and are less accurate for geometric alteration [31]. Periapical radiographs are still widely used, especially in Indonesia, therefore the use of periapical radiographs in the analysis of osseointegrating implants should be further investigated.

A visual periapical radiograph assessment for each phase of bone healing in the osseointegration process is not visible. However, the potential and advantages of radiographs encourage further analysis of radiograph capabilities. Therefore, this research aimed to analyze the differences in the osseointegration image of the dental implant using periapical radiographs with density and bone morphometry parameters (trabecular thickness, separation, and number) on three stages of implant osseointegration: day 3, 14, and 28.

## 2. Materials and Methods

### 2.1. Surgery Procedure and Radiographic Evaluation

This experimental research was conducted at Dental Radiology Installation, Dental and Oral Hospital, Padjadjaran University, Bandung, Indonesia, and the research protocol was approved by the Animal Ethics Committee, Faculty of Veterinary Medicine, Bogor, Indonesia (006/KEH/SKE/III/2021). Furthermore, this research was carried out by FP for 3 months from November 2021 to January 2022. The population consisted of a New Zealand white rabbit (*Oryctolagus cuniculus*). The periapical radiographs were obtained from the tibia bone.

In this research, 12 rabbits aged 6 months (weight 3.0–3.5 kg) were used as a sample. The adaptation period for the rabbits lasted for 2 weeks. The rabbits were kept in their cages and received laboratory standard feed once a day, with tap water chow ad libitum. The rabbit fur was shaved and the skin surface was cleaned with an iodine solution. The rabbits were anesthetized with a combination of 10 mg/kg of ketamine hydrochloride (Pharmamadix Corp, Peru) and 3 mg/kg of xylazine hydrochloride (Interchemie werken “De Adelaar” BV, Venray, Holland) intramuscular. An incision was carried out on a 2 cm muscle on the superior proximal tibia, then the muscles were dissected with an artery clamp. The dental implant used was a tapered type (Dentium Co. Ltd., Korea), made of titanium alloy and coated by SA (sunblasted with alumina acid). The implant size was 4 mm in diameter and 7 mm in length.

Implant surgeries were performed by an experienced surgeon with a standardized protocol with slight modification [32]. The implant installation was performed following the manufacturer's recommendation. The lance drill with 800–1200 rpm was used to penetrate the cortical bone as an implant insertion site, then a twist drill (7 mm length). The depth of the hole and the bottom condition of the hole are checked using a depth gauge and checking the position and direction of the hole using a parallel pin. The drilling sequence was started with pilot drill (diameter), twist drill 2, 3, 4 mm diameters was used sequentially in low-speed drill at 50–60 rpm, and then ended by attaching implant with mount driver. The implant was covered with an appropriate cover screw and the muscle was sutured layer-by-layer with nonabsorbable material [33]. After implant installation, antibiotic amoxicillin long-acting 15 mg/kg and analgetic flunixin 2.5 mg/kg were injected through intramuscular and the rabbit was returned to their cages. The rabbit was divided into three groups based on the healing time: day 3, 14, and 28.

**Figure 1:**
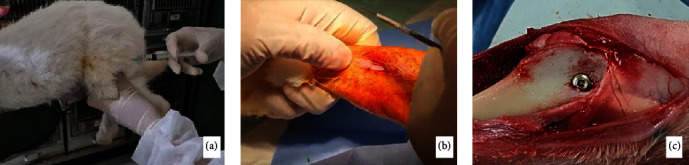
The installation of implants in the tibia of rabbits: (a) the rabbit was anesthetized via intramuscular injection; (b) incision on the superior proximal tibia; (c) implant installation.

The rabbits were euthanized with an overdose of 30 mg/kg of ketamine hydrochloride (Pharmamadix Corp, Peru) and 9 mg/kg of xylazine hydrochloride (Interchemie werken “De Adelaar” BV, Venray, Holland) by intramuscular injection. The tibia was dissected about 1.0 cm in length from the implant's outer surface with a low-speed carborundum disk. All dissected bone segments were fixed in a 10% neutral-buffered formalin solution. The installation of implants in the tibia of rabbits is shown in Figure 1.

The periapical radiographs measurement was carried out at three different healing times; day 3, 14, and 28. The conventional X-ray equipment Digital X-ray Reader A4/Transparent scanner with 1,200 dpi resolution was operated at 60 kV, 7 mA, an exposure time of 0.16 s, and a focus-receptor distance of 9 cm. All periapical photographs were carried out with the parallel technique.

### 2.2. Imaging Processing

The 12 periapical radiographs data consist of four data sets for each of the three stages of implant osseointegration: day 3, 14, and 28 (*n* = 12). The periapical radiographs should have a complete image covering all parts of the dental implant (from coronal to apical), have a good image quality, and have the implant in an upright position. Image quality criteria are visually assessed using seven methods; object completeness, contrast, density, sharpness, detail, distortion, and brightness. If a periapical radiograph contains data with metal artifacts, beam hardening, and the existence of streaks, the sample is excluded. The overall radiographs were saved and extracted in the standard digital imaging and communications in medicine (DICOM) format of the Cliniview software (Cliniview Software, Finland). The variables observed were density and Tb.Th., Tb.Sp., and Tb.N. on day 3, 14, and 28.

**Figure 2:**
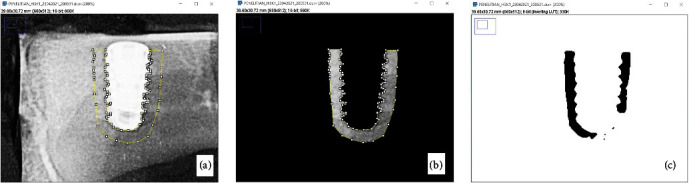
The density analysis and bone morphometry imaging process: (a) ROI selection on periapical radiographs; (b, c) digital processing on ImageJ cropping-filter Gaussian blur-thresholding.

Density analysis and bone morphometry were performed using ImageJ software version 1.53c (National Institutes of Health, US) by selecting the BoneJ plugin on a computer (Toshiba Portege Intel Core 13, Tokyo, Japan) with Windows 7 (Microsoft, Washington, USA) [34, 35]. Measurement of density analysis and bone morphometry for each periapical radiograph were performed by an intraobserver test. The observers repeated the evaluation three times in a 2-week interval [36]. The morphometric measurement started with converting all the data from DICOM to 64-bit format and then determining the region of interest (ROI) using the free-hand selection tool. Furthermore, the ROI was created according to criteria such as the outer contour area of the dental implant thread from the apex, base, and lateral, followed by a line covering the osseointegrated or trabecular area. The ROI was identified by tool-free hand selection on 1 mm a width around the implant. The image preprocessing process used the Gaussian filter blur adjustment to smooth the objects and adjusted the threshold to clarify the edges of the trabeculae examining the dark background. This converted the image into a binary shape, the trabeculae were separated from the nontrabeculae or bone marrow. The density analysis and bone morphometry imaging process can be seen in Figure 2. The density and bone morphometry analysis was carried out in triplet for each measurement time (day 3, 14, and 28) and variable (density, Tb.Th., Tb.Sp., and Tb.N.).

### 2.3. Statistical Analysis

Statistical analysis was performed by SPSS version 18 (SPSS, Chicago, Illinois, USA) software. The result was presented by the mean and standard deviation (SD), also the median and range. The significant difference was evaluated to identify the difference in the density, Tb.Th., Tb.Sp., and Tb.N. values for each measurement time. The significant difference of normally distributed data was evaluated by analysis of variance (*F*-test) with *p* < 0.05. Furthermore, the significant difference in the non-normally distributed data was evaluated by the nonparametric Kruskal–Wallis test with *p* < 0.05. The data from four variables were compared using Spearman's correlation with *p* < 0.05 confidence interval.

## 3. Results

A total of 12 periapical radiographs were selected based on inclusion and exclusion criteria. The 12 periapical radiographs consist of four data sets for every three stages of implant osseointegration, which are day 3, 14, and 28. The one periapical radiograph was analyzed in four parameters; Density, Tb.Th., Tb.Sp., and Tb.N.

Table 1 shows the descriptive statistical data from measurement results three times. Table 1 shows the normality test data for each parameter on day 3, 14, and 28. The normality test is used to determine whether the sample data have been taken from a normally distributed population, which is shown by a *p*-value > 0.05.

Based on Table 1, the value of density, Tb.Th., and Tb.Sp. for each treatment time showed a *p*-value higher than 0.05. It is indicated that density, Tb.Th., and Tb.Sp. data on day 3, 14, and 28 were normally distributed. The *p*-value for the Tb.N. parameter on day 3 and 14 showed a value higher than 0.05; however, the *p*-value on day 28 showed a value of 0.028 (<0.05). Based on Tb.N. *p*-value, the data of Tb.N. were not normally distributed.

Table 2 shows the comparison of examination results of density, Tb.Th., Tb.Sp., and Tb.N., and the significant difference *p*-value on day 3, 14, and 28. The significant difference of normally distributed data (density, Tb.Th., and Tb.Sp.) was evaluated by analysis of variance (*F*-test). The comparative measurements of not normally distributed data (Tb.N.) used the nonparametric Kruskal–Wallis test analysis.

Based on Table 2, density and Tb.Th. show a *p*-value < 0.05, which are 0.042 and 0.08, respectively. However, the Tb.Sp. shows a value of *p*-value > 0.05 which is 0.086. Based on the *F*-test, density and Tb.Th. show a significant difference between day 3, 14, and 28. Unfortunately, the *F*-test shows that between day 3, 14, and 28 of Tb.Sp. value had no significant difference. Based on the Kruskal–Wallis test, the Tb.N. values on day 3, 14, and 28 showed a *p*-value > 0.05, which was 0.164. It is indicated that the Tb.N. values on day 3, 14, and 28 had no significant difference.

**Figure 3:**
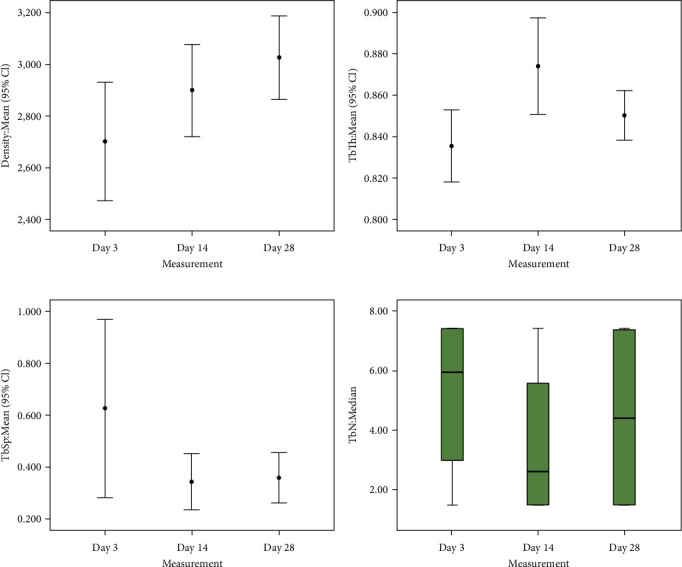
Comparison of (a) density, (b) Tb.Th., (c) Tb.Sp., and (d) Tb.N. values of osseointegration examination results from the three measurement times: (a) the density data are illustrated as mean + standard deviation; (b) the Tb.Th. data are illustrated as mean + standard deviation; (c) the Tb.Sp. data areere illustrated as mean + standard deviation; (d) the Tb.N. data are illustrated as median and range.

The results of each variable on day 3, 14, and 28 are shown in Figure 3. Figure 3 shows the trend density, Tb.Th., Tb.Sp., Tb.N. on day 3, 14, and 28.

Based on Figure 3(a), the density value shows an increasing trend in treatment time manner. However, the values of Tb.Th., Tb.Sp., and Tb.N. do not show the treatment time manner trend. The Tb.Th. value increased from day 3 to 14 and decreased on day 28 (Figure 3(b)). In contrast, Tb.Sp. and Tb.N. values show the same trend. The values of Tb.Sp. (Figure 3(c)) and Tb.N. (Figure 3(d)) showed a decrease from day 3 to 14 and increased on day 28.

The comparison between density, Tb.Th., Tb.Sp., and Tb.N. can be seen in Table 3. The correlation of density among Tb.Th., Tb.Sp., and Tb.N. are showed a Spearman's correlation (*ρ*) of 0.500 or moderate. However, the *p*-value of the correlation is 0.667, which indicates that the density have no significant correlation to Tb.Th., Tb.Sp., and Tb.N. values.

Based on Table 3, there was a significantly very strong and negative correlation between Tb.Th. and Tb.Sp., also Tb.Th. and Tb.N., in which *ρ* is 1.000 and *p*-value is 0.001. A negative correlation was shown as min (−), and it is indicated that high value of Tb.Th. frequently occur with low value of Tb.Sp. and Tb.N. The Tb.Sp. value has a significant very strong and positive correlation with the Tb.N. value, in which *ρ* is 1.000 and *p*-value is 0.001. Positive correlation is shown as plus (+), and it is indicated that high value of Tb.Sp. tends to coincide with high value of Tb.N.

## 4. Discussion

The postimplant radiography is specialized to view the reaction around the implant, taken at intervals from the beginning of the placement and continued for as long as clinically needed [37]. Evaluation of the image of the supporting bone, such as the volume and quality of the surrounding, is an important step in the planning and success of dental implant treatment. This research was conducted to determine the differences in the osseointegrated image of the dental implant using periapical radiographs in terms of density, and evaluation of Tb.Th., Tb.Sp., and Tb.N. on day 3, 14, and 28. Furthermore, the parameters used were measurements extracted from the archive of periapical radiographs at the Dental and Oral Hospital, Padjadjaran University. The four variables were examined to represent the osseointegration process of the dental implant on day 3, 14, and 28.

This research used the superior proximal tibia as the implantation site. The proximal tibia is an excellent source of cancellous bone and several previous studies have suggested the tibia for oral surgical reconstruction [38, 39]. The great volume of the tibia and ease of operation have made it possible to use this bone for the production of peri-implant defects [40], an examination of bone regeneration related to dental implants [41], and the use of spacers and a bone substitute model [42]. Previous research showed that harvesting of the cancellous bone from the proximal tibia allows early mobilization of the patient after the operation for an average of 20.59 days [43]. Moreover, it was confirmed by Atil [44] that all transplanted tibial autogenous bone grafts were well integrated 100% into the recipient sites. The tibia has a fast-healing process and the osseointegration can be analyzed 1 month after implantation [45], and it has satisfactory long-term stability and a good tissue healing capacity [46].

The results show a significant change in the density value (*p* < 0.05) on day 3, 14, and 28. The success of implant installation is indicated by an increase in the density value during osseointegration assessment. According to Colnot et al. [47] and Berglundh et al. [48], the process on day 3 is an early stage of bone formation. The previous report was in line with the present research (Table 2) that density value increased significantly from day 3, 14, to 28 based on statistical calculation. This condition indicates that the 3rd day after implant placement is still in the inflammatory process. On the radiographs, this process is shown in the form of a radiolucent area around the implant [30].

The next process is proliferation, which starts with the growth of fibrous tissue and ends with less mineralized woven bone up to mineralized lamellar bone [49]. Ramachandran et al. [50] reported an increase in bone density, which was evaluated after 3–6 months in the apical lateral of the implant through a pixel assessment (grayscale). This is in line with the research, which shows an increase in the density value starting from day 3 to 28. The increased density is probably due to the growth of fibrous tissue into bone tissue. The fibrous tissue formed undergoes calcification with the deposition of osteoblasts to form a lamellar layer [11]. The bone healing process ends with bone remodeling around the implant occurring ∼1 month after implant placement [51].

The results (Table 2) show that the trabecular thickness variable increases, especially on day 14. This is due to a process of bone maturation in each implant installed. After day 28, the medullary canal and trabeculae appear thickened due to the growth of lamellar bone fibers in the area of the dental implant interface [52]. The thickening of trabeculae in this research is in line with the osseointegration timing in dental implants. Furthermore, this research shows a process of bone formation that begins in the 2nd week, as evidenced by a change in the radiographic image from radiolucent to radio intermediate. This is in line with Franchi et al.'s [53] investigation, which examined the femur of sheep and stated that the process of bone deposition would occur continuously. The trabecular bone fills the gap between the implant and the tissue starting on day 14 [53].

The next bone morphometries are trabecular separation and trabecula number. Based on Table 2, the trabecular separation result shows a maximum value on day 3 and decreases on day 14 and 28. This is because there is bone growth around the implant, and De Lange et al. [54] stated that the bone healing process lasted for several weeks. This condition results in a change in the radiographic appearance around the implant, initially radiolucent to radiopaque [30]. The loss of the radiolucent image indicates that the space under the implant is filled with bone [54]. Meanwhile, the trabecular number (Tb.N.) shows insignificant results where the value changes on day 3, 14, and 28 do not follow a pattern. The healing process from the inflammation and proliferation is directly proportional to the trabecular number, which is in line with the value of osteoblasts [11].

The comparison of density was not significantly correlated to Tb.Th., Tb.Sp., and Tb.N. (Table 3). Muller stated that bone density measurements should be followed by trabecular bone morphometry because the measurements of the two characters sometimes show different values [55]. Other studies have explained that high-density values do not match the trabecular parameters, which show no increase in trabecular number (Tb.N.) and thickness (Tb.Th.) [56]. Previous statement was in line with the present study result that density and the trabecular bone morphometry (Tb.Th., Tb.Sp., and Tb.N.) show the different value.

However, the assessment of bone quality after a dental implant seems to be subject to some limitations. In this research, the sample size is based on the previous investigation, because it considers the time and cost factors. In further research, a power analysis in the calculation of the sample size is required for more reasonable statistical power and more reliable results. Moreover, the analysis of bone quality after dental implant can be examined for a larger population and more appropriate normalization parameters can be added. This research used periapical radiographs modalities for assessing the osseointegration process after implantation. It is showing no pattern of an increase in the trabecula number due to the limitations of the radiographs analyzed by periapical radiographs. Moreover, changes in the digitization of the radiographs reduce the quality and therefore many data cannot be analyzed. Based on that, the using periapical radiograph to evaluate osseointegration is a limitation in this research. To complete the research for information or clinical decision-making, further investigations are suggested to use histopathological analysis to analyze stages of osteointegration.

## 5. Conclusion

This research demonstrates a healing process after implant placement evidenced by new bone growth from day 3, 14, and 28 as three stages of implant osseointegration on the assessment of periapical radiographs. The bone density and the trabecular thickness (Tb.Th.) show the significant difference for among three-stage implant osseointegration. However, trabecular separation (Tb.Sp.) and trabecular number (Tb.N.) showed no significant difference for each three-stage implant osseointegration.

## Figures and Tables

**Table 1:** Descriptive statistics data from measurement results three times.

Variable	Statistical size				Data normality test (*p*-value ^*∗*^)
	Mean	SD	Median	Range	
1. Density					
Day 3	2,701.42	359.796	2,664.5	2,329–3,151	0.368
Day 14	2,898.67	280.793	2,989.5	2,331–3,256	0.611
Day 28	3,024.67	253.58	2,968.5	2,826–3,802	0.629
2. Tb.Th.					
Day 3	0.836	0.027	0.826	0.811–0.881	0.322
Day 14	0.874	0.037	0.887	0.819–0.921	0.553
Day 28	0.850	0.019	0.840	0.834–0.881	0.080
3. Tb.Sp.					
Day 3	0.625	0.538	0.375	0.25–1.50	0.051
Day 14	0.344	0.170	0.375	0.125–0.50	0.224
Day 28	0.358	0.152	0.375	0.15–0.50	0.131
4. Tb.N.					
Day 3	5.188	2.567	5.930	1.48–7.41	0.272
Day 14	3.520	2.533	2.600	1.47–7.41	0.205
Day 28	4.421	3.077	4.396	1.47–7.41	**0.028**

Note:  ^*∗*^The data were measurement in three times. The normality value below 0.005 is shown in bold. The density, Tb.Th., and Tb.Sp. data are mean (SD), while Tb.N. data are median and range.

**Table 2:** Comparison of examination results using periapical radiographs.

Variable	Measurement time			*p*-value ^*∗*^
	D-3	D-14	D-28	
1. Density	2,701.42	2,898.67	3,024.67	**0.042**
2. Tb.Th.	0.836	0.874	0.850	**0.008**
3. Tb.Sp.	0.625	0.344	0.358	0.086
4. Tb.N.	5.930	2.600	4.396	0.164

Note:  ^*∗*^With the *F*-test (analysis of variance), except for Tb.N. with the Kruskal–Wallis test. The *p*-value below 0.005 is shown in bold. The density, Tb.Th., and Tb.Sp. data are mean (SD), while Tb.N. data are median and range.

**Table 3:** Comparison between four variables from periapical radiograph.

Variable	Density	Tb.Th.	Tb.Sp.	Tb.N.
Density					
*ρ*	1.000	0.500	−0.500	−0.500
Sig. (2-tailed)	0.001	0.667	0.667	0.667
Tb.Th.				
*ρ*	0.500	1.000	−1.000^*∗*^	−1.000^*∗*^
Sig. (2-tailed)	0.667	0.001	0.001	0.001
Tb.Sp.				
*ρ*	−0.500	−1.000^*∗*^	1.000	1.000^*∗*^
Sig. (2-tailed)	0.667	0.001	0.001	0.001
Tb.N.				
*ρ*	−0.500	−1.000^*∗*^	1.000^*∗*^	1.000
Sig. (2-tailed)	0.667	0.001	0.001	0.001

Note: The correlation analysis used Spearman rank's correlation. *ρ*, correlation coefficient.  ^*∗*^Correlation is significant at *p* < 0.05.

## Data Availability

The data used to support the findings are available from the corresponding author upon request.
